# Acenocoumarol, an Anticoagulant Drug, Prevents Melanogenesis in B16F10 Melanoma Cells

**DOI:** 10.3390/ph16040604

**Published:** 2023-04-17

**Authors:** Hyunju Han, Changgu Hyun

**Affiliations:** Jeju Inside Agency and Cosmetic Science Center, Department of Chemistry and Cosmetics, Jeju National University, Jeju 63243, Republic of Korea; 00guswn00@naver.com

**Keywords:** acenocoumarol, B16F10, drug repurposing, melanogenesis, signal pathways

## Abstract

Hyperpigmentation can occur in abnormal skin conditions such as melanomas, as well as in conditions including melasma, freckles, age spots, seborrheic keratosis, and café-au-lait spots (flat brown spots). Thus, there is an increasing need for the development of depigmenting agents. We aimed to repurpose an anticoagulant drug as an effective ingredient against hyperpigmentation and apply cosmeceutical agents. In the present study, the anti-melanogenic effects of two anticoagulant drugs, acenocoumarol and warfarin, were investigated. The results showed that both acenocoumarol and warfarin did not cause any cytotoxicity and resulted in a significant reduction in intracellular tyrosinase activity and melanin content in B16F10 melanoma cells. Additionally, acenocoumarol inhibits the expression of melanogenic enzymes such as tyrosinase, tyrosinase-related protein (TRP)-1, and TRP-2, suppressing melanin synthesis through a cAMP-dependent, protein kinase (PKA)-dependent downregulation of microphthalmia-associated transcription factor (MITF), a master transcription factor in melanogenesis. Furthermore, anti-melanogenic effects were exerted by acenocoumarol through downregulation of the p38 and JNK signaling pathway and upregulation of extracellular signal-regulated kinase (ERK) and phosphatidylinositol 3 kinase (PI3K)/protein kinase B (Akt)/glycogen synthesis kinase-3β (GSK-3β) cascades. In addition, the β-catenin content in the cell cytoplasm and nucleus was increased by acenocoumarol through a reduction in the phosphorylated β-catenin (p-β-catenin content). Finally, we tested the potential of acenocoumarol for topical applications by conducting primary human skin irritation tests. Acenocoumarol did not induce any adverse reactions during these tests. Based on the results, it can be concluded that acenocoumarol regulates melanogenesis through various signaling pathways such as PKA, MAPKs, PI3K/Akt/GSK-3β, and β-catenin. These findings suggest that acenocoumarol has the potential to be repurposed as a drug for treating hyperpigmentation symptoms and could provide new insights into the development of therapeutic approaches for hyperpigmentation disorders.

## 1. Introduction

Drug repurposing is the process of identifying new uses for existing drugs that were originally developed for a different therapeutic indication. This strategy involves exploring the potential of already approved drugs to treat diseases or conditions other than those they were initially intended for [1,2]. The concept of drug repurposing is based on the observation that many drugs have multiple biological effects and mechanisms of action, which may be useful for treating different diseases [3,4]. By repurposing existing drugs, researchers can bypass the time-consuming and costly process of drug discovery and development, which can take up to a decade and cost billions of dollars [1,2,3,4]. Repurposing gained renewed attention during the pandemic after the FDA granted emergency use authorizations (EUAs) for several repurposed drugs to treat COVID-19 [5,6,7]. Many small-molecule inhibitors and activators against human diseases have been identified through drug repurposing. The nature of skin diseases, particularly hyperpigmentation, makes the development of effective anti-melanogenic agents challenging. Herein, we examined the applicability of the anti-coagulant acenocoumarol in drug repositioning for hyperpigmentation.

**Figure 1:**
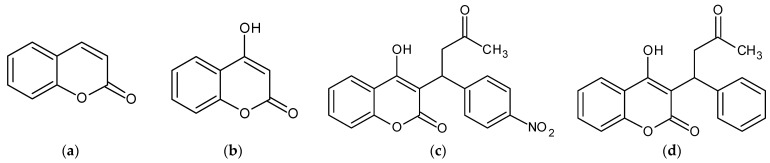
Structure of coumarin derivatives. (**a**) Coumarin, (**b**) 4-hydroxycoumarin, (**c**) acenocoumarol, and (**d**) warfarin.

Acenocoumarol is an anticoagulant drug that is derived from 4-hydroxicoumarin. It is similar in structure to warfarin but has a nitro group at the 4’ position. Acenocoumarol is widely used as an oral anticoagulant, second only to warfarin. It works as a vitamin K antagonist by inhibiting vitamin K epoxide reductase, which affects the carboxylation of coagulation factors [8,9]. Acenocoumarol is not approved for use by the U.S. Food and Drug Administration in the United States, but it is available in other countries such as Canada. Acenocoumarol may be a potential drug for inducing the differentiation of glioma cells using a drug repositioning strategy, and its mechanism of action has been reported to involve histone deacetylation, DNA methylation, and retinoic acid signaling pathways [10]. As shown in Figure 1, 4-Hydroxycoumarin is a derivative of coumarin, with a hydroxyl group at position 4. It is commonly used as a starting material in organic synthesis [11]. Its derivatives are attracting attention because of their properties as oral anticoagulants, antibiotics, antifungal, antiviral, antioxidant, anti-inflammatory, antiprotozoal, insecticidal, antimutagenic agents, and tyrosine kinase inhibitors [12,13,14]. The topography of 4-hydroxycoumarin reveals that it has both electrophilic and nucleophilic properties. The carbon atom at position 3 exhibits the most prominent reactivity, which is attributed to its nucleophilic nature [15]. Therefore, it has been widely used for its synthetic endpoints, and also for its special biological activities. Acenocoumarol and its analog warfarin are both 4-hydroxycoumarin derivatives and are commonly used as anticoagulants to prevent thromboembolic diseases such as infarction and transient ischemic attacks, and for the management of conditions such as deep vein thrombosis and myocardial infarction [16]. Furthermore, acenocoumarol has been found to inhibit tryptophan breakdown in Caco-2 cells that were stimulated with IFN-γ in a dose-dependent manner [17]. In our efforts for drug repurposing with potent and safe skin health effects, we focused on the anti-coagulants acenocoumarol and warfarin. Our preliminary study revealed these two anti-coagulants as the most potent anti-melanogenic agents. In this study, a new approach was used to investigate the anti-melanogenic effects of acenocoumarol through the regulation of the MAPK, PKA/CREB, and PI3K/Akt/GSK-3β signaling pathways using a B16F10 cell model. Additionally, the potential of acenocoumarol as a topical anti-melanogenic agent was tested using a human skin primary irritation test.

## 2. Results

### 2.1. Acenocoumarol and Warfarin Reduced Melanin Synthesis and Tyrosinase Activity in B16F10 Cells

**Figure 2:**
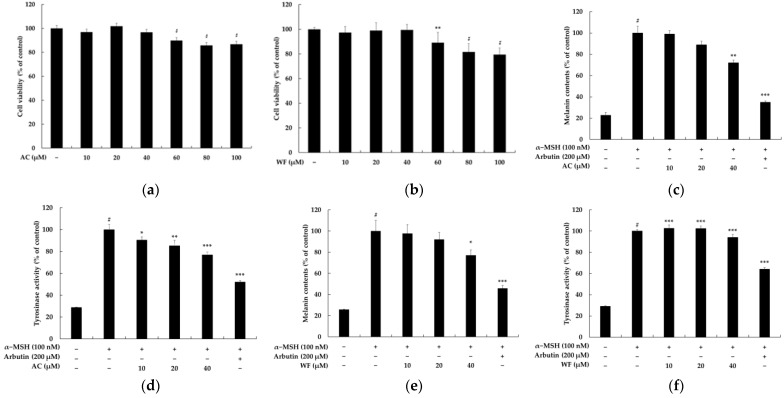
The effect of coumarin derivatives on the viability, melanin contents, and tyrosinase activity in B16F10 melanoma cells. The cells were treated with coumarin derivatives (10, 20, 40, 60, 80 and 100 μM) for 72 h. Cytotoxicity of acenocoumarol (**a**, AC) and warfarin (**b**, WF) were evaluated using the MTT assay. Cell viability is expressed as percentages relative to untreated cells. The results are presented as the mean ± SD from three repeated experiments. For melanin production and tyrosinase activity, the cells were treated with AC (**c**,**d**) and WF (**e**,**f**) for 72 h at 10, 20 and 40 μM concentration. α-MSH was used as the negative control and arbutin (200 μM) was used as the positive control. We conducted three repeated measurements using Image J and present the results as mean ± SD. Statistical significance was expressed as follows: # *p* < 0.001 vs. unstimulated control group, * *p* < 0.05, ** *p* < 0.01, *** *p* < 0.001 vs. α-MSH alone.

**Figure 3:**
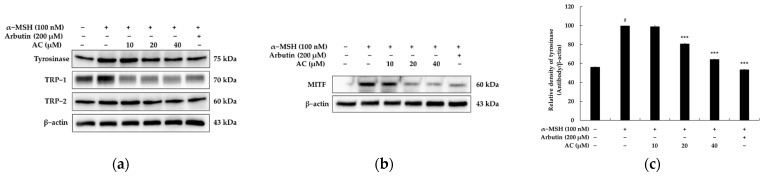

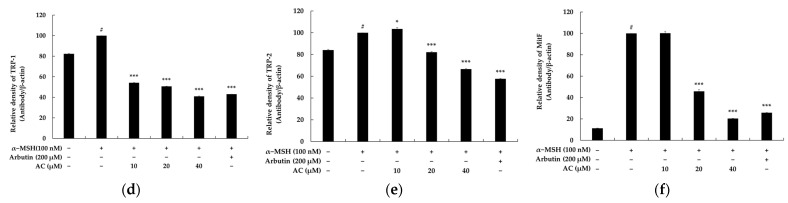
The effect of acenocoumarol on tyrosinase, TRP-1, TRP-2, and MITF protein expression in α-MSH-stimulated B16F10 cells. α-MSH was used as the negative control and arbutin (200 μM) was used as the positive control. (**a**,**b**) Protein expression levels of TYR, TRP-1, TRP-2, and MITF were determined by Western blot following with acenocoumarol (10, 20 and 40 μM) treatment for 48 h in the presence of α-MSH (100 nM). The densitometric analysis of TYR (**c**), TRP-1 (**d**), TRP-2 (**e**), and MITF (**f**) using Image J and present the results as mean ± SD. Statistical significance was expressed as follows: # *p* < 0.001 vs. unstimulated control group, * *p* < 0.05, *** *p* < 0.001 vs. α-MSH alone.

The chemical structures of acenocoumarol and warfarin are shown in Figure 1. In this study, we investigated the potential cytotoxicity of acenocoumarol and warfarin against B16F10 melanoma cells. Our findings indicate that both compounds did not exert any toxicity up to a concentration of 40 μM; however, there was a slight effect on cell viability at the 60 μM concentration (Figure 2a,b). Therefore, subsequent experiments were conducted using concentrations lower than 60 μM. As shown in Figure 2c,e, acenocoumarol and warfarin demonstrated a concentration-dependent reduction in melanin content. Additionally, the activity of tyrosinase was also reduced in cells pretreated with acenocoumarol and warfarin (Figure 2d,f). Further experiments were conducted to evaluate the anti-melanogenic effects of acenocoumarol, as the compound showed a greater inhibition of melanin content and tyrosinase activity compared to warfarin (Figure 3).

### 2.2. Acenocoumarol Regulated the Expression of Melanogenic Proteins

Three important melanogenic enzymes, tyrosinase, tyrosinase-related protein (TRP)-1, and TRP-2, are required for the proper production of melanin synthesis. The study investigated the mechanisms by which acenocoumarol inhibits melanogenesis in B16F10 cells. The expression of three enzymes involved in melanogenesis, namely tyrosinase, TRP-1, and TRP-2, is regulated by a master regulator, MITF [18,19]. The study found that acenocoumarol decreased the protein expression of tyrosinase, TRP-1, TRP-2, and MITF in B16F10 cells, indicating that the inhibition of melanogenesis by acenocoumarol is mediated by the downregulation of these enzymes via MITF (Figure 3).

### 2.3. Acenocoumarol Inhibited Melanogenesis through the PKA Signaling Pathway

**Figure 4:**
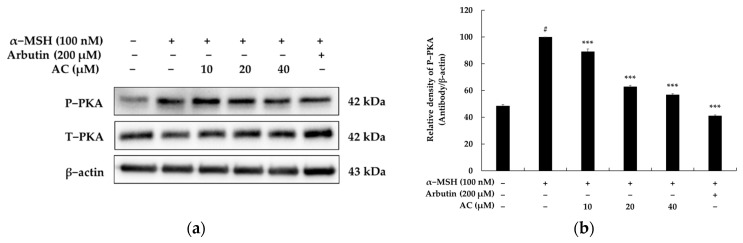
The effect of acenocoumarol on PKA protein expression in α-MSH-stimulated B16F10 cells. The cells were treated with acenocoumarol (10, 20 and 40 μM) for 24 h in the presence of α-MSH (100 nM). (**a**) Western blotting results, (**b**) P-PKA protein expression. α-MSH was used as the negative control and arbutin (200 μM) was used as the positive control. We conducted three repeated measurements using Image J and present the results as mean ± SD. Statistical significance was expressed as follows: # *p* < 0.001 vs. unstimulated control group, *** *p* < 0.001 vs. α-MSH alone.

The expression of the MITF gene, which is mediated by PKA signaling, upregulates the expression of critical factors in melanogenesis, including tyrosinase, TRP-1, and TRP-2, in a sequential manner [20]. Therefore, having demonstrated that acenocoumarol can induce the expression of MITF, tyrosinase, TRP-1, and TRP-2, we further investigated whether the melanogenic activity of acenocoumarol involves the PKA signal in B16F10 cells by performing Western blotting. As shown in Figure 4, acenocoumarol treatment significantly downregulated the expression level of phosphorylated PKA as compared with the control treatment. Thus, the above findings suggest that acenocoumarol-induced down-regulation of melanogenesis in B16F10 cells may be, at least in part, mediated through activation of the cAMP/PKA pathway.

### 2.4. Acenocoumarol Suppressed Melanogenesis via the MAPK Signaling Pathway

**Figure 5:**
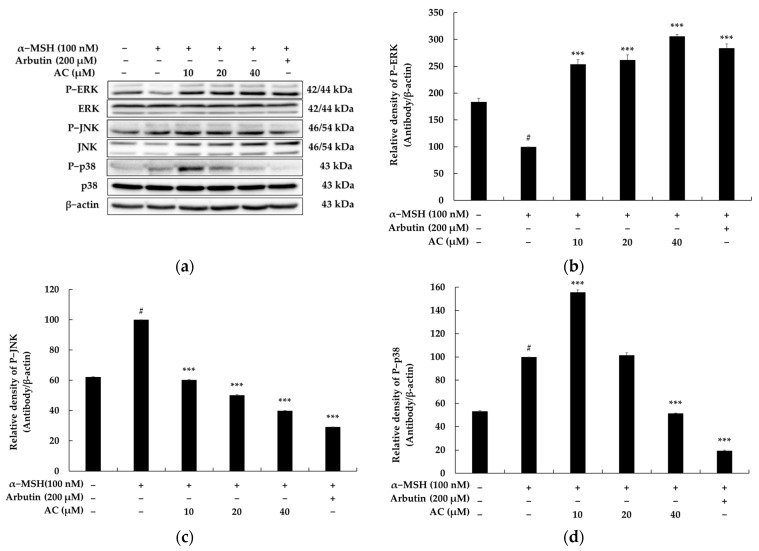
The effect of acenocoumarol on MAPKs protein expression in α-MSH-stimulated B16F10 cells. The cells were treated with acenocoumarol (10, 20 and 40 μM) for 4 h in the presence of α-MSH (100 nM). Western blotting results (**a**), P-ERK protein expression (**b**), P-JNK protein expression (**c**), P-p38 protein expression (**d**). α-MSH was used as the negative control and arbutin (200 μM) was used as the positive control. We conducted three repeated measurements using Image J and present the results as mean ± SD. Statistical significance was expressed as follows: # *p* < 0.001 vs. unstimulated control group, *** *p* < 0.001 vs. α-MSH alone.

Previous research has shown that the phosphorylation of mitogen-activated protein kinases (MAPKs) controls the expression of MITF. It has also been found that inhibiting the phosphorylation of p38 and JNK, while activating ERK phosphorylation, leads to reduced expression of MITF and melanogenic enzymes, resulting in the downregulation of melanogenesis [21]. Hence, we assessed the phosphorylation of p38, ERK, and JNK MAPKs to study the upstream cascade related to the anti-melanogenesis effect of acenocoumarol. As illustrated in Figure 5, treatment with acenocoumarol resulted in a marked decrease in the levels of phosphorylated p38 and JNK, and a significant reduction in ERK phosphorylation compared to the control treatment. Based on these findings, it can be inferred that acenocoumarol exerts anti-melanogenic effects on melanoma cells by reducing MITF expression via the MAPK signaling pathway.

### 2.5. Acenocoumarol Repressed Melanogenesis through PI3K/Akt/GSK-3β Signaling Pathways

**Figure 6:**
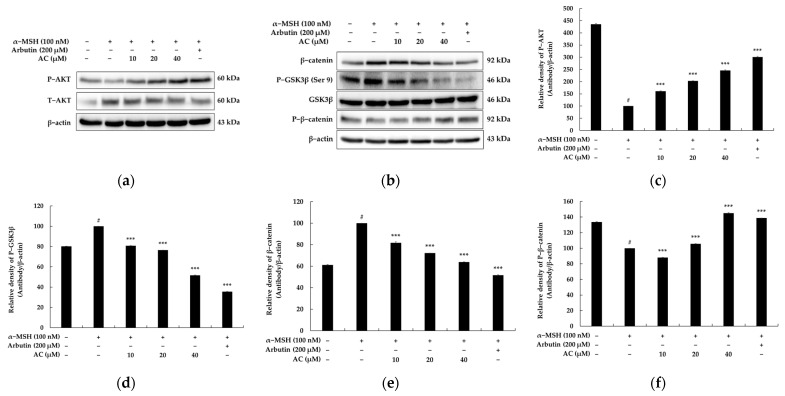
The effect of acenocoumarol on AKT, P-GSK3β, β-catenin, and P-β-catenin protein expression in α-MSH-stimulated B16F10 cells. α-MSH was used as the negative control and arbutin (200 μM) was used as the positive control. (**a**,**b**) Protein expression levels of AKT, P-AKT, GSK3β, P-GSK3β, β-catenin, and P-β-catenin were determined by Western blot following with acenocoumarol (10, 20 and 40 μM) treatment for 4 h in the presence of α-MSH (100 nM). The densitometric analysis of P-AKT (**c**), GSK3β (**d**), β-catenin (**e**), and P-β-catenin (**f**) using Image J and present the results as mean ± SD. Statistical significance was expressed as follows: # *p* < 0.001 vs. unstimulated control group, *** *p* < 0.001 vs. α-MSH alone.

The activation of the PI3K/Akt pathway inhibits the transcriptional activity of MITF for melanogenic proteins, leading to the suppression of melanin synthesis. Furthermore, it has been shown that GSK-3β phosphorylation by PI3K/Akt induces MITF degradation and inhibits melanogenesis [22]. Therefore, we investigated whether acenocoumarol inhibits melanogenesis through the activation of the PI3K/Akt/GSK-3β pathways in α-MSH-treated B16F10 cells. As illustrated in Figure 6, Western analyses revealed that the treatment of acenocoumarol on α-MSH-stimulated B16F10 cells for 48 h led to an increase in the phosphorylation levels of Akt, and a decrease in GSK-3β levels. These results suggest that the inhibitory effect of acenocoumarol on melanogenesis is linked to the PI3K/Akt/GSK-3β pathways.

### 2.6. Acenocoumarol Repressed Melanogenesis through Wnt/β-Catenin Signaling Pathways

Recently, Wnt/β-catenin signaling has been recognized as a crucial regulator involved in melanin synthesis [23]. To determine whether the Wnt/β-catenin signaling pathway is involved in acenocoumarol-mediated anti-melanogenesis, we examined the expression of β-catenin in α-MSH-treated B16F10 cells. Earlier studies have demonstrated that the phosphorylation of GSK3β (Ser 9) in the Wnt/β-catenin pathway causes the accumulation of β-catenin in the cytoplasm. Subsequently, this leads to an increase in the expression of MITF in the nucleus [23]. We examined whether acenocoumarol reduces melanogenesis in B16F10 cells by targeting the Wnt/β-catenin signaling pathway. The results indicate that acenocoumarol increases the levels of P-GSK3β (Ser 9) and β-catenin as compared to the untreated group. However, acenocoumarol inhibits the expression of P-β-catenin as compared to the untreated group. These findings suggest that acenocoumarol reduces melanogenesis by targeting the Wnt/β-catenin signaling pathway (Figure 6).

### 2.7. Acenocoumarol Was Found to Be a Safer Ingredient though a Human Primary Irritation Test

We carried out primary human skin irritation tests to evaluate the potential of acenocoumarol for topical application. We applied concentrations of 20 and 40 µM of acenocoumarol to patches of skin for up to 24 h and observed the patches 20 min and 24 h after removing the acenocoumarol. As a negative control, we used squalene (solvent). The results presented in Table 1 show that the test substance (acenocoumarol) was categorized as “causing no to slight irritation” in terms of its primary irritation potential on human skin.

## 3. Discussion

Skin pigmentation disorders are a group of conditions caused by the under- or overproduction of melanin in the skin, resulting in a significant healthcare burden, especially for patients of color. Hyperpigmentation disorders, such as melasma and post-inflammatory hyperpigmentation, are particularly problematic because there are few safe and effective treatments. The small molecule hydroquinone, currently the only approved treatment for hyperpigmentation in the United States, is under regulatory review due to its possible carcinogenicity, and its use has recently been severely restricted in the United States and other countries [24]. Therefore, a demand exists for new effective drugs that inhibit the overproduction of melanin in the skin. Similar to hydroquinone, most photosensitizers inhibit tyrosinase, a copper-containing oxidase inside melanocytes that catalyzes the initial steps of melanin synthesis. Novel tyrosinase inhibitors have been discovered through high-throughput screening of small-molecule libraries. Mechanistically, most of them act as competitive inhibitors, competing with Y for the active site [25]. Other drug development strategies include repurposing existing drugs or finding new uses for diseases for which they were not initially developed. Repurposing established drugs is a way to quickly find more effective treatments, which can be used to stabilize pandemics or treat rare diseases. Such drug repurposing has been promoted extensively over the past few years, as it is a rapid drug development approach able to bring effective drugs to the market for a variety of indications [26,27]. The field of cosmeceuticals targeting the skin is no exception: skin appearance is essential for self-esteem and quality of life, and therefore skincare products represent a huge market. In particular, cosmeceuticals are a hybrid category of skin care formulations that are halfway between cosmetics and pharmaceuticals and are rationally designed to target (pathological) physiological mechanisms to improve skin health and appearance [28,29,30].

Cosmeceuticals are used for anti-aging, anti-wrinkles, hair regrowth, skin whitening, and wound healing, with a particular emphasis on scarless healing. The development of all these commercial cosmeceuticals, including inhibitors of skin pigmentation disorders, has greatly accelerated, along with our approach to selecting drug compounds. By selecting substances that have already been tested and proven safe, we can make new cosmeceutical products more quickly and cheaply [31].

Acenocoumarol effectively and safely treats atrial fibrillation, cardiac valve replacement, deep vein thrombosis, and other conditions in all age groups. It is a mono-coumarin derivative with a racemic mixture of R (+) and S (−) enantiomers. Its efficacy and safety have undergone evaluation after myocardial infarction, major surgeries, and critical illness requiring prolonged hospitalization [16]. It has been reported to possess diverse biological properties, such as antitumor, antibiotic, and anti-inflammatory activities [9,10,11,12,13]. However, to our knowledge, no reports investigating the effect of acenocoumarol on melanogenesis have been published. Therefore, the present study was conducted to evaluate the potential of acenocoumarol as a hypopigmenting agent for cosmetic applications. There is a standard process for such studies. The first step is to test whether the agent inhibits melanin production in a model system such as B16F10 cells, and the second step is to test whether it inhibits the production of tyrosinase, TRP-1 and TRP-2, proteins involved in melanogenesis, and whether it inhibits the production of MITF, a transcription factor that regulates these melanogenesis-related enzymes. There are various signaling pathways that regulate the transcription factor MITF, including the cAMP–CREB–PKA pathway, MAPK pathways, PI3K/Akt/GSK-3β pathways, and Wnt/β-catenin pathways [18,19,20,21,22,23]. Therefore, by proving the involvement of these signaling pathways, we can secure the reliability of the skin hypopigmenting agents.

The inhibition of melanogenesis induced by acenocoumarol and warfarin is not likely due to its nonspecific toxic effect since the viability of melanocytes treated with acenocoumarol and warfarin remains unchanged. We found that acenocoumarol and warfarin significantly reduced melanin synthesis within the safe concentration range (10–40 μM) by inhibiting the rate-limiting enzyme tyrosinase, without causing any harm to the B16F10 cells. Figure 3 demonstrated that acenocoumarol had a stronger effect on inhibiting melanin content and tyrosinase activity than warfarin. Acenocoumarol is a hydroxycoumarin similar to warfarin in which the hydrogen at position 4 of the phenyl substituent is replaced by a nitro group. These subtle structural differences seem to affect melanin production and tyrosinase activity in an α-MSH-induced mouse B16F10 model system. Although the process of melanin biosynthesis is complex, recent evidence suggests that MITF plays a crucial role in the pigmentation process of melanocytes. It is important to note that MITF regulates the expression of the melanogenic enzymes TYR, TYR-1, and TYP-2 during melanin synthesis [32]. Our results revealed that acenocoumarol treatment suppressed the MITF expression, which resulted in a decrease in melanogenic enzymes in hormone-induced mouse B16F10 cells.

It has been reported that UV exposure can increase PKA activity by promoting the secretion of α-MSH. PKA activation then leads to the upregulation of CREB phosphorylation, which positively regulates MITF expression. Consequently, CREB phosphorylation ultimately stimulates the elevation of MITF expression. To understand the mechanisms underlying the acenocoumarol-induced reduction in MITF expression, we assessed the protein levels of PKA, a key transcription factor involved in MITF expression [33,34]. Our study findings demonstrate that acenocoumarol treatment suppresses PKA activation, which results in the downregulation of MITF expression. These outcomes imply that acenocoumarol can inhibit melanin synthesis by inhibiting MITF through PKA/CREB signaling cascades, subsequently reducing the expression levels of TYR, TRP-1, and TRP-2 in α-MSH-induced B16F10 cells. We next conducted a Western blot assay to investigate the molecular mechanisms of acenocoumarol on melanogenesis in B16F10, focusing on important MAPK pathway proteins. Previous studies have shown the crucial role of MAPK signaling pathways in melanin synthesis, including P38, JNK, and ERK1/2. Upon phosphorylation, p-P38 and p-JNK activate downstream MITF expression, whereas p-ERK1/2 induces MITF phosphorylation, leading to MITF degradation via ubiquitin-dependent pathways [21]. Our results showed decreased phosphorylation levels of P38 and JNK and increased phosphorylation levels of ERK1/2 in acenocoumarol-treated cells. This suggests that the increased phosphorylation of P38 and JNK protein could lead to more inhibition of MITF protein production and phosphorylation, while the decreased p-ERK1/2 could result in more MITF degradation. Consequently, the final MITF protein and phosphorylation level was significantly decreased, contributing to inhibited melanogenic protein expression. Although the melanin contents and cellular tyrosinase activity did not decrease significantly, the significant decrease in MITF expression can reflect the decreased melanogenic protein expression of acenocoumarol, as many melanin synthesis signaling pathways eventually converge to MITF. It is worth noting that the role of MAPK pathway activation in melanin production is still a subject of debate. In B16 melanoma cells, schisandrin B has been shown to inhibit melanin production by suppressing the phosphorylation levels of ERK, JNK, and p38 MAPK.

The regulation of melanin synthesis is a complex process, partly because phosphorylation can both increase the transcriptional activity of MITF and induce its degradation through ubiquitin proteasome-dependent mechanisms [35,36]. It is interesting to note that the PI3K/AKT and GSK3β pathways appear to have opposing effects on melanogenesis. While inhibition of PI3K increases melanin synthesis, inhibition of GSK3β also increases melanogenesis. Acenocoumarol was shown to induce GSK3β phosphorylation, which is critical for its downstream effects on melanogenesis. This suggests that the PI3K/AKT-GSK3β axis mediates the effects of acenocoumarol on melanogenesis. Furthermore, recent studies have shown that AKT can directly induce MITF phosphorylation at Ser510, leading to its degradation by proteases [21]. This could explain why the PI3K inhibitor induced more melanin synthesis than the GSK3β inhibitor.

**Figure 7:**
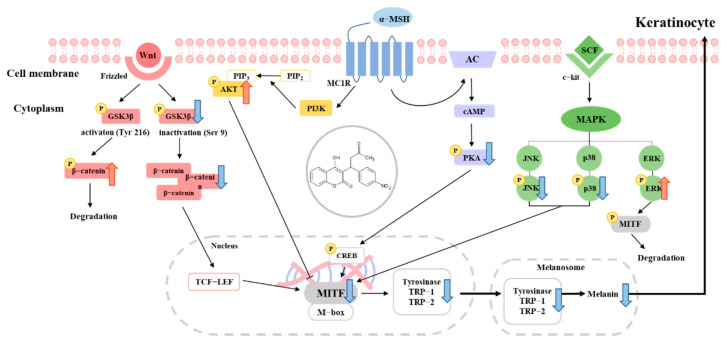
Schematic diagram of the proposed mechanism regulating the inhibitory action of acenocoumarol on melanogenesis.

The Wnt signaling pathway is also involved in the regulation of MITF expression. Activation of the canonical Wnt pathway leads to the inactivation of GSK3β and accumulation of β-catenin, which translocates into the nucleus and enhances the expression of MITF. Therefore, inhibiting GSK3β can promote melanogenesis. Conversely, activated GSK3β (phosphorylated at Tyr216) can induce the phosphorylation and degradation of β-catenin, leading to decreased expression of MITF [37,38,39]. The present findings suggest that acenocoumarol inhibits MITF expression in part by decreasing the inactive form of GSK3β and increasing the active form, leading to decreased levels of β-catenin in the nucleus and inhibition of MITF transcription. Overall, these results suggest that the Akt/GSK3β/β-catenin signaling pathway plays an important role in the regulation of melanogenesis. Inhibition of this pathway by acenocoumarol may contribute to the suppression of MITF expression and the antimelanogenesis effect observed in the study (Figure 7).

Finally, we conducted primary human skin irritation tests to determine whether acenocoumarol could potentially be used as a topical material. They tested concentrations of 20 or 40 μM acenocoumarol on the skin of 31 healthy volunteers to assess any stimulation or sensation potential. Acenocoumarol was deemed to cause “no to slight irritation” in this analysis. These results suggest that using acenocoumarol as a topical agent may prevent the pathogenesis of pigmentation disorders. However, the potential involvement of the signaling pathways mentioned above in inhibiting melanin synthesis through acenocoumarol requires further investigation in the future. Although we demonstrated the melanogenic effects of acenocoumarol in B16F10 melanoma cells, the relative effectiveness of acenocoumarol in human normal melanocytes is yet to be determined in future studies. Furthermore, it is necessary to evaluate the efficacy and safety of acenocoumarol-induced melanogenesis inhibition in animal and human models.

## 4. Materials and Methods

### 4.1. Materials

TCI (Tokyo, Japan) supplied the acenocoumarol used in this study, while Sigma-Aldrich (St. Louis, MO, USA) provided the warfarin. Thermo Fisher Scientific (Waltham, MA, USA) supplied penicillin-streptomycin, Dulbecco’s modified Eagle’s medium (DMEM), NE-PER nuclear and cytoplasmic extraction reagents, 0.5% Trypsin-EDTA (10×), and BCA protein assay kit, and Merck Millipore (Burlington, MA, USA) supplied fetal bovine serum (FBS). Sigma-Aldrich (St. Louis, MO, USA) provided sodium hydroxide (NaOH), L-DOPA, protease/phosphatase inhibitor cocktail, and α-melanocyte-stimulating hormone (α-MSH). Biosesang (Seongnam, Gyeonggi-do, Korea) supplied dimethyl sulfoxide (DMSO), 3-(4,5-dimethylthiazol-2-yl)-2,5-diphenyltetrazolium bromide (MTT), tris-buffered saline (TBS), sodium dodecyl sulfate (SDS), phosphate-buffered saline (PBS), radioimmunoprecipitation assay (RIPA) buffer, and the enhanced chemiluminescence (ECL) kit. BD Difco (Sparks, MD, USA) supplied skim milk, and Bio-Rad (Hercules, CA, USA) provided tween 20 and 2× laemmli sample buffer. We purchased the primary antibodies for tyrosinase (SC-20035), MITF (SC-71588), TRP-1 (SC-166857), and TRP-2 (SC-74439) used for Western blotting from Santa Cruz Biotechnology (Dallas, TX, USA). Additionally, we purchased the secondary anti-mouse and anti-rabbit antibodies, as well as p-PKA (5661S), p-ERK (9101S), p-p38 (9211S), PKA (4782S), p-JNK (9251S), ERK (9102S), p38 (9212S), JNK (9252S), p-AKT (9271S), AKT (9272S), p-GSK-3β (9322S), GSK-3β (5676S), p-β-catenin (9561S), β-catenin (25362S), and β-actin (4967S) antibodies from Cell Signaling Technology (Danvers, MA, USA).

### 4.2. Cell Culture

The mouse melanoma B16F10 cells were purchased from ATCC: The Global Bioresource Center (Manassas, VA, USA). B16F10 cells were grown as monolayers in a humidified atmosphere at 37 °C with 5% CO_2_, using media supplemented with DMEM with 10% FBS and 1% penicillin-streptomycin.

### 4.3. MTT Assay

We conducted an MTT assay to assess cytotoxicity. We treated B16F10 cells (1.5 × 10^4^ cells/well) with acenocoumarol in 24-well plates and incubated them for 72 h. To perform the MTT assay, we replaced the culture medium with MTT (0.2 mg/mL, 500 μL) and incubated the cells at 37 °C for 3 h. We then removed the medium and dissolved the formazan product in DMSO. Finally, we measured the absorbance at 570 nm using a microplate reader (Biotek; Winooski, VT, USA).

### 4.4. Measurement of Melanin Contents

We incubated B16F10 cells (8.0 × 10^4^ cells/dish) in 60 mm cell culture dishes for 24 h. Then, we treated the cells with acenocoumarol and α-MSH (100 nM) and cultured them for 72 h. We used arbutin (200 μM) as a positive control. After washing the cells with 1 × PBS, we added RIPA lysis buffer and lysed them at 4 °C for 15 min. We then centrifuged the lysates for 20 min at 12,000 rpm and −8 °C to remove the supernatant and obtain a pellet. We dissolved the cell pellets in 1 N NaOH supplemented with 10% DMSO at 90 °C for 10 min. Finally, we measured the absorbance at 405 nm using a microplate reader (Biotek; Winooski, VT, USA).

### 4.5. Measurement of Tyrosinase Activity

We estimated the tyrosinase activity by measuring the rate of L-DOPA oxidation. We incubated B16F10 cells (8.0 × 10^4^ cells/dish) in 60 mm cell culture dishes for 24 h. Then, we treated the cells with coumarin derivatives and α-MSH (100 nM) and cultured them for 72 h. We used arbutin (200 μM) as a positive control. After washing the cells with 1 × PBS, we added RIPA lysis buffer and lysed them at 4 °C for 15 min. We then centrifuged the lysates for 15 min at 12,000 rpm and −8 °C to obtain the supernatants. We quantified the protein concentration at 30 μg/mL using a BCA protein assay kit. Next, we added L-DOPA (2 mg/mL) to the quantified protein and incubated it at 37 °C for 1 h. Finally, we measured the absorbance at 490 nm using a microplate reader (Biotek; Winooski, VT, USA).

### 4.6. Western Blot Analysis

We mixed protein and 2 × Laemmli sample buffer in a 1:1 ratio and heated it at 100 °C for 5 min to prepare the loading sample. Then, we used SDS-polyacrylamide gel electrophoresis to separate the proteins by size. The protein was then transferred to a PVDF membrane and blocked in 5% skim milk dissolved in TBS-T for 2 h. After washing the membrane with 1 × TBS-T, we reacted it overnight at 4 °C with the primary antibody dissolved in a ratio of 1:2000. We then washed the antibody and reacted the membrane with the secondary antibody dissolved in a ratio of 1:1000 at room temperature for 2 h. Finally, we expressed the protein using an ECL kit and developed it using a ChemiDoc (Vilber Lourmat, Marne La Vallée, France).

### 4.7. Primary Skin Irritation Test

For this study, we recruited 31 healthy female volunteers aged between 20 and 60 years who had no prior history of irritant and/or allergic contact dermatitis. The mean age of the participants was 43.19 ± 5.97 years, and their ages ranged from 29 to 53 years. We prepared a negative control, which was squalene-added imperatorin, and applied it at concentrations of 20 and 40 μM. We assessed the primary skin irritation responses according to the PCPC guidelines and calculated the reaction results for each test substance using the formula shown below. We conducted the study with the written consent of each volunteer, in compliance with the Helsinki Declaration’s ethical principles for medical research. The Industrial Review Board (IRB) of Dermapro Inc. approved the study (IRB number: 1-220777-A-N-01-DICN22080).

$$\mathrm{Response}=\frac{\sum \left(Grade\times No.\;of\;Responders\right)}{4\;\left(Maximum\;Grade\right)\times n\;\left(Total\;Subjects\right)}\times 100\times 1/2$$

### 4.8. Statistical Analyses

We conducted three repeated experiments, and the results are expressed as the mean and standard deviation (mean ± SD). The Student’s *t*-test was used to express statistical significance as a *p*-value. The unstimulated control group had a p-value of less than 0.001. The α-MSH or LPS alone groups had *p*-values of * *p* < 0.05, ** *p* < 0.01, and *** *p* < 0.001.

## Figures and Tables

**Table 1:** The results from the primary human skin irritation tests (*n* = 31).

No.	Test Sample	No. of Respondents	20 min after Removal				24 h after Removal				Reaction Grade (R) *		
			+1	+2	+3	+4	+1	+2	+3	+4	24 h	48 h	Mean
1	Acenocoumarol n (20 μM)	0	-	-	-	-	0	-	-	-	0	0	0
2	Acenocoumarol (40 μM)	1	-	-	-	-	1	-	-	-	0	1	0.4
3	Squalene	0	-	-	-	-	-	-	-	-	0	0	0

* The investigator evaluated the reactions 20 min and 24 h after removing the treatment, following the PCPC guidelines (2014). The range of irritation was classified as “no to slight irritation” with values ranging from 0.00 to less than 0.87.

## Data Availability

Data is contained within the article.
